# A Misdiagnosed Case of Hypertrophic Gastropathy

**DOI:** 10.1155/2020/4562531

**Published:** 2020-12-01

**Authors:** Sushma Thapa, Arnab Ghosh, Gita Pun, Dilasma Ghartimagar, O. P. Talwar

**Affiliations:** ^1^Department of Pathology, Manipal College of Medical Sciences, Pokhara, Nepal; ^2^Department of Pathology, Gandaki Medical College and Teaching Hospital, Pokhara, Nepal

## Abstract

Hypertrophic gastropathy is a rare idiopathic hyperproliferative disorder which may present as Menetrier's disease (MD) characterized by foveolar hyperplasia in the gastric fundus and body. It is often accompanied by a severe loss of plasma proteins (including albumin) from the altered gastric mucosa. The disease occurs in two forms, a childhood form due to cytomegalovirus infection and an adult form attributed to overexpression of transforming growth factor-alpha (TGF-*α*). The most common symptoms include epigastric pain with fullness and vomiting and generalized peripheral edema with hypoalbuminemia. We present a case of 75-year-old female presenting with epigastric pain and vomiting. Upper gastrointestinal endoscopy and computed tomography scan revealed an irregular mucosal fold at the body and antrum and thickening of the stomach wall, respectively. Though the endoscopic gastric mucosal biopsy was nonspecific, the patient underwent partial gastrectomy due to clinicoradiological suspicion of carcinoma. On histopathology, the case was reported as hypertrophic gastropathy, consistent with MD. Though there is a strong clinical and radiological suspicion of malignancy in the hypertrophied gastric mucosa, MD should be one of the important differential diagnoses.

## 1. Introduction

Hypertrophic gastropathy is recognized by three basic conditions, viz., (a) Menetrier's disease—hyperplasia of the foveolar layer, (b) Zollinger-Ellison Syndrome (ZES)—hyperplasia of the glandular layer, and (c) combined foveolar-glandular hyperplasia [1]. Menetrier's disease (MD) is a rare disease that was first described by the French pathologist Pierre Menetrier in 1888 [2]. It is an unusual acquired hypertrophic gastropathy leading to the dilation of the mucus-secreting gastric pits (foveola) along with atrophy of the gastric glands producing acid and pepsinogen. Due to these changes, the disease is characterized by the huge expansion of the gastric mucosa, thick mucus secretion, protein loss, and hypochlorhydria [3]. The disease is more common in male (3 : 1) and between the fourth and sixth decade of age [4]. The disease occurs in two forms, a childhood form due to cytomegalovirus infection and an adult form attributed to overexpression of transforming growth factor-alpha (TGF-*α*) [5].

## 2. Case Presentation

**Figure 1:**
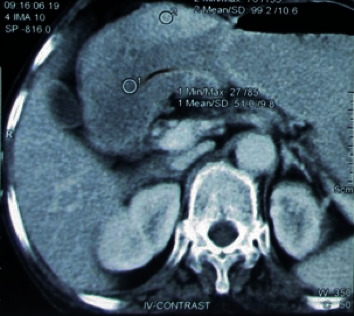
Axial computed tomography image of the abdomen showing a thickened wall of the stomach at the body and pyloric end.

**Figure 2:**
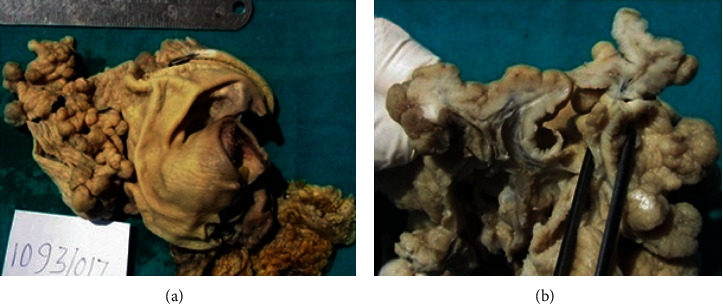
(a) Gross specimen of the stomach with multiple polypoidal lesions in the body with a normal-looking surrounding mucosa. (b) Cut section through polypoidal lesions showing mucosal thickening not extending beyond the submucosa.

**Figure 3:**
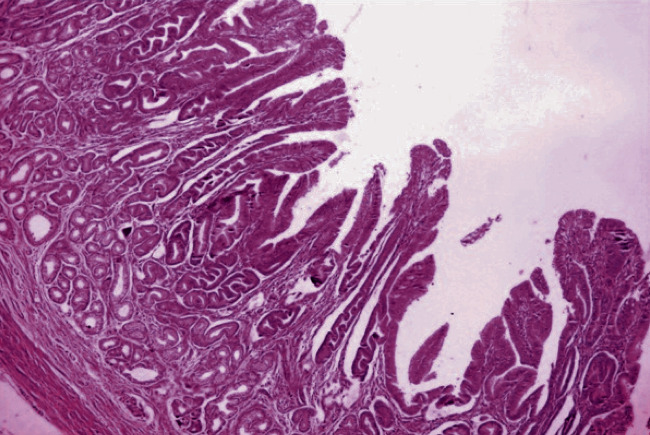
Photomicrograph of the thickened gastric mucosa showing a corkscrew type of foveolar hyperplasia with elongation and tortuosity along with atrophy of the underlying gastric glands (H&E, ×400).

A 75-year-old female, from a remote hilly area, housewife by occupation, presented with epigastric pain and vomiting on and off for 5-month duration. The patient also had a history of swelling of the lower limb on and off. There was no relevant family or past medical and surgical history. The general appearance of the patient was fair. All the vitals were within normal limits, and there was no lymphadenopathy. On systemic examination, she had a distended abdomen with no hepatosplenomegaly or ascites. All the blood parameters, viz., complete blood count, random blood sugar, renal function test, liver function test, and serum electrolytes, were within normal limit. The serum albumin was within normal limit (40 g/L). Stool for occult blood was negative. The serological tests for HIV, HBsAg, and HCV were also normal. Ultrasonography (USG) of the abdomen showed thickening of the wall of the stomach with luminal narrowing. Upper gastrointestinal (UGI) endoscopy reported an exophytic growth at the body of the stomach with a hard base. A contrast-enhanced computed tomography (CECT) scan of the abdomen also showed thickening of the wall of the stomach at the body and pyloric end reaching up to the first part of the duodenum, and carcinoma of the stomach was suggested (Figure 1). An endoscopic biopsy was done, and chronic gastritis was reported with no evidence of *H. pylori*. Due to radiological suspicion of carcinoma of the stomach, the patient underwent exploratory laparotomy with partial gastrectomy. Grossly, the specimen consisted of part of the stomach measuring 12 cm in length with an attached omentum measuring 18 × 6 cm. The mucosal surface showed multiple polypoidal lesions involving a 9 × 4 cm area at the body of the stomach (Figure 2(a)). Another single polypoidal lesion was also identified at the distal end measuring 1 × 1 cm. On the cut section, the lesion was seen as thickened gray white and glistening mucosal infoldings which were not extending beyond the submucosa (Figure 2(b)). Histologically, the sections showed a hypertrophied gastric mucosa with a corkscrew type of foveolar hyperplasia with elongation and tortuosity along with atrophy of the underlying gastric glands. Some of the glands were cystically dilated. The lamina propria was edematous and infiltrated by chronic inflammatory cell infiltrates comprising of lymphocytes, plasma cells, and eosinophils. There was no evidence of atypia (Figure 3). The case was reported as hypertrophic gastropathy, consistent with MD. Currently, on a 20-month follow-up, the patient is in good health condition.

## 3. Discussion

In 1888, Menetrier described the terms “Polyadenomes polypeux” equivalent to multiple hyperplastic polyps and “Polyadenomes en nappe” in which Menetrier's disease is restricted at present. It is also known as hypertrophic or hyperplastic gastropathy, giant hypertrophic gastritis, and giant hypertrophy of gastric rugae [6]. It is a rare idiopathic hyperproliferative disorder of the foveolar mucus surface epithelium of the gastric fundus and body associated with hypoproteinemia and edema [7].

MD is an extremely rare disease, and only a few hundred cases have been reported in the literature [3, 8]. It most commonly affects adults between the fourth and sixth decade of age with male predilection (3 : 1) which however differs from the present case [4]. The adult form is attributed to overexpression of transforming growth factor-alpha (TGF-*α*) whereas the childhood form is linked to infection with cytomegalovirus [5]. In the present case, an appropriate test for the cytomegalovirus was not available in our hospital. Morphological cellular changes were also not seen in the histological section of the resected specimen suggesting the infection with cytomegalovirus. And moreover, the age of the patient in this case was 75 years.

Clinically, the disease is insidious with a progressive clinical course and classically present with gastrointestinal symptoms, peripheral edema, and giant gastric folds. The most common gastrointestinal symptoms include epigastric pain, anorexia, weight loss, and vomiting which are similar to those of the present case study. Abnormal enteric protein loss is manifested by hypoalbuminemia and generalized peripheral edema [7]. For the diagnosis of MD, hypoalbuminemia is considered a cornerstone, but similar to other studies, our case had albumin levels within normal ranges despite having peripheral edema [9].

Radiological studies include a barium swallow of upper gastrointestinal series with small bowel follow through or a CECT scan which often shows diffuse thickening of the gastric wall often sparing the antrum in contrast to our study where the CECT scan and UGI endoscopy revealed an irregular mucosal fold and thickening of the stomach wall involving both the body and antrum [7]. USG of the abdomen also revealed the similar findings. With various radiological modalities, definitive diagnosis cannot be made with certainty and hyperplastic gastritis, gastric malignancy, gastric polyposis syndrome, and ZES have to be ruled out [3].

In the present case, an endoscopic pinch biopsy could not establish the diagnosis. Because accurate diagnosis of some of these diseases including MD requires examination of the very thick gastric mucosa, large snare biopsies that capture the entire thickness of the mucosa are recommended instead of standard forceps biopsies [10]. Sanchez et al. [11] reported a case of MD in which diagnosis was made by an invasive laparoscopic-assisted full-thickness biopsy after failure of multiple endoscopic pinch trials. Several studies have shown an association of *H. pylori* with MD [12, 13], and many authors proposed that *H. pylori* plays an important role in the pathogenesis process of MD [14]. However, similar to our case, Azer et al. and several other studies reported hypertrophic gastropathy or MD without demonstrable *H. pylori* [9, 15, 16].

The gastrectomy specimen of the present case had multiple polypoidal growth with thickened gray white and glistening mucosal infoldings which were not extending beyond the submucosa. Juvenile polyposis and other polyposis syndromes sometimes involve the stomach and may mimic MD which may be differentiated by family history, manifestations outside of the stomach, genetic testing, appearance at endoscopy, and histological presentation [3]. In a case series of 48 patients done by Rich et al. for the evaluation for possible MD, 3 were diagnosed with juvenile polyposis, 1 with Cronkhite-Canada Syndrome, and 4 with other hamartomatous polyps or an uncharacterized polyposis syndrome [3].

Histological diagnosis of MD is based on the features of a significant increase in the height of the mucus cell compartment of the gastric mucosa with giant hyperplasia of the foveolar layer, with polyp-like formations and unusually deep tortuous and often cystically dilated foveolus along with reduced glands as observed in our case [4]. The histological differential diagnosis includes gastritis polyposa profunda and hyperplastic chronic active, lymphocytic, or allergic gastroenteritis [7]. Lack of inflammatory cells is the key to differentiate MD from lymphocytic or allergic hypertrophic gastritis and *Helicobacter pylori* infection. Diffuse thickening of the foveolae by the tortuous hyperplastic epithelium is the key to differentiate MD from the localized changes predominantly at the base of the glands in gastritis polyposa profunda [17, 18]. MD is distinguished histologically from hyperplastic polyps and juvenile polyps by the preservation of tissue architecture and parallelism of gastric glands and the presence of prominent lamina propria smooth muscle fibres [10]. Hyperplastic polyps and juvenile polyps will appear more disorganised and edematous. The other histological differential diagnosis also includes ZES which is characterized by a thickened mucosa of the gastric body, with measurement of up to 5-8 mm [18], due to parietal cell hyperplasia which also extends to the base of the glands, in zones normally occupied by chief cells. The foveolar compartment is not expanded [10].

Medical treatment of the disease includes proton pump inhibitors, high protein diet, eradication of *H. pylori* [14], cetuximab (monoclonal antibody) [19], and octreotide long-acting release [16]. However, gastrectomy is a well-established treatment of hypertrophic gastropathy or MD due to the high rate of failure of medical treatment and the risk of malignant transformation [15]. In our case, though the endoscopic gastric biopsy was performed, it could not establish the diagnosis and the patient underwent partial gastrectomy due to clinicoradiological suspicion of carcinoma.

## 4. Conclusion

Hypertrophic gastropathy is a rare idiopathic hyperproliferative disorder which may present as MD characterized by foveolar hyperplasia in the gastric fundus and body associated with gastrointestinal symptoms, hypoproteinemia, and edema. Despite having strong clinical and radiological suspicion of malignancy, MD should be one of the differentials of the hypertrophied gastric mucosa with or without *H. pylori* or hypoalbuminemia.

## Data Availability

N/A
